# The Discrepancy of Parents’ Theories of Intelligence and Parental Involvement

**DOI:** 10.3389/fpsyg.2019.01231

**Published:** 2019-06-06

**Authors:** Kexin Jiang, Juan Liu, Chunhui Liu, Xiaolin Guo, Huan Zhou, Bo Lv, Zhaomin Liu, Liang Luo

**Affiliations:** ^1^Collaborative Innovation Center of Assessment toward Basic Education Quality, Beijing Normal University, Beijing, China; ^2^School of Sociology, China University of Political Science and Law, Beijing, China; ^3^Collaborative Innovation Center of Assessment toward Basic Education Quality and Institute of Developmental Psychology, Beijing Normal University, Beijing, China

**Keywords:** parental involvement, parents’ congruence and discrepancy, polynomial regression with response surface analysis, theory of intelligence, Chinese parents

## Abstract

In families, mothers and fathers may hold the same or different levels of theories of intelligence. This congruence and discrepancy may influence parental involvement in children’s education. The current study examined how both parents’ theories of intelligence and the direction and degree of the discrepancy of parents’ intelligence theories influence maternal and paternal involvement separately. We measured 1,694 matched pairs of parents’ theories of intelligence and educational involvement, and examined the relationships using linear regressions and polynomial regressions with response surface analysis. The results showed that (1) the mother’s intelligence theory positively related to both paternal involvement and maternal involvement, but the father’s intelligence theory only positively related to paternal involvement; (2) when the parents’ theories of intelligence reached congruence, the parents’ theories of intelligence are positively related to both maternal and paternal involvement; (3) when the parents’ theories of intelligence have discrepancy, the maternal involvement is higher while the mother’s intelligence theory’s level is more incremental than father’s; and (4) when the parents’ theories of intelligence have discrepancy, more discrepancy of parents’ theories of intelligence is related to more paternal involvement. This study revealed the significance of mother’s role in education, highlighted the importance of parents’ congruence and discrepancies in beliefs, examined how parents’ beliefs impact their own behavior and their couple’s behavior.

## Introduction

The theory of intelligence (implicit theory of intelligence, intelligence mindset) (Dweck and Leggett, 1988) refers to beliefs that people hold concerning the nature of intelligence, namely, the changeability of intelligence (Hong et al., 1999). Specifically, there are two main types of theories of intelligence: the incremental theory and the entity theory. The incremental theory assumes that intelligence is malleable and changeable, most notably through effort and persistence, while the entity theory assumes that intelligence is fixed and not easily changed (Dweck and Leggett, 1988). Based on empirical research, Walker et al. (2005) put forward a theoretical model, considering that the parent’s theory of intelligence was an important psychological factor influencing parental involvement.

Parental involvement, namely, parents’ engagement in their children’s education, is a variety of behaviors that parents perform to promote their children’s academic achievement and psychological development in their homes and schools (Seginer, 2006). Theoretical studies such as the models by Walker et al. (2005) and Hornby and Lafaele have described the link between parental theory of intelligence and parental involvement. These studies have contended that, on the one hand, parents who hold an incremental theory of intelligence most likely emphasize the role of effort, motivate children to accept shortcomings, encourage them to think about the mechanisms underlying specific questions, and are more involved in education. On the other hand, parents who hold an entity theory of intelligence believe strongly in the preeminence of ability over effort and often lack confidence, which leads to actions that minimize external judgments. Furthermore, these parents regard children’s difficulties with learning as reflecting low ability, which leads to decreased parental involvement (Walker et al., 2005; see the model of the barriers to parental involvement, Hornby and Lafaele, 2011). However, those models only hypothesized such a relationship.

Researchers have used data to provide evidence for the close relationship between parental theory of intelligence and parental involvement. Studies have shown that primary-school students’ parents who hold more incremental theories reported higher frequency of engagement in math- and reading-related activities with their children (Muenks et al., 2015); mothers who view their children’s performance as more important than learning were more likely to choose easy activities for their children than were mothers who view learning as more important (Ames and Archer, 1987; Stipek et al., 1992); mothers who were induced to hold an entity theory displayed a higher frequency of unconstructive involvement (Aunola et al., 1999; Moorman and Pomerantz, 2010).

However, although previous research has revealed the relationship between the parent’s theory of intelligence and parental involvement from both theoretical and empirical perspectives and helped us understand why there are individual differences in involvement frequency, these studies are still insufficient. First, the studies above concentrated only on the relationship between one parent’s theory of intelligence and his or her own involvement in the child’s learning. However, an individual’s behavior is influenced not only by his or her own beliefs but also by the environment. According to the family system theory, the family is a complete system (an emotional unit), and family members are a component of that system. Consequently, every member of the family mutually interacts with others (Bowen, 1966, 2010). Previous studies have also supported this interaction. For example, a study of 622 dual-earner mothers in the United States demonstrated that mothers’ global expectations and beliefs about the allocation of family work and their recognition of the father’s educational ability influenced paternal involvement (maternal gatekeeping; Allen and Hawkins, 1999). Zvara et al. (2013) discovered that fathers demonstrated greater direct engagement in their child’s healthcare when mothers held more nontraditional beliefs about gender roles. Based on this evidence, we can draw the conclusion that paternal and maternal involvement relates to each parent’s theory of intelligence; however, until now, no studies have examined whether both parents’ theories of intelligence together relate to one parent’s involvement.

Second, the abovementioned studies have neglected the effect of the congruence and discrepancy of parents’ theories of intelligence on paternal and maternal involvement. Obviously, there are individual differences in theories of intelligence. Therefore, there might be a discrepancy in parents’ theories of intelligence within a family (Bosma et al., 1996); in one family, both parents may hold incremental theories at the same level, whereas in another family, the father may hold an incremental theory while the mother holds an entity theory. Accordingly, there may be a substantial difference between the two families with regard to the parental involvement modes. We can reasonably assume that parents’ theories at different levels might negatively affect their motivation to be involved in their children’s education, and high incremental theories held by both parents might lead to a higher level of involvement in the family. Conversely, complete congruency can also be a problem (Carlson et al., 1991). If both parents believe that intelligence is unchangeable, they may be unwilling to be involved in their children’s learning, which is worse than if one parent believes that intelligence can be changed. Some studies in similar fields have provided secondary evidence for this situation. One study focused on family members’ (father, mother, adolescent) perceptions and beliefs about the nature of autonomy and its development and on their degree of congruence or discrepancy within the family (Cicognani and Zani, 1998). Another two studies examined how parents’ discrepancies in childrearing beliefs impact coparenting (Egeren and Hawkins, 2004; McHale et al., 2004). However, the details regarding the impact of the congruence or discrepancy of parents’ theories of intelligence on paternal and maternal involvement remain to be revealed.

In addition to the two issues above, in China, both parental involvement and the theory of intelligence might have particular connotations because of the cultural context. Evidence has shown that Chinese parents place an exceptionally high value on education and are actively engaged in their children’s education at home (Zhang and Carrasquillo, 1996; Lizza and Huang, 2008; Huntsinger and Jose, 2009; Wang and Gao, 2013), which contributes to the frequency of parental involvement. Furthermore, within collectivist culture, Chinese parents are more likely to encourage students to learn existing knowledge rather than to create new things when tutoring their child in their studies (Lizza and Huang, 2008; Shao and Zhang, 2010). In terms of the theory of intelligence, in cross-cultural studies, researchers have found that Chinese parents always pay more attention to competing and obtaining good grades, while Western parents emphasize the individual’s growth, encouraging students to compare their achievement only with themselves (Tobin et al., 1989; Zhao, 2005; Li, 2007), which means that more Chinese parents might hold entity theories rather than incremental theories. Consequently, researching the relationship between the parent’s theory of intelligence and parental involvement in the Chinese context can allow us to better understand the differences and changes in the relationship within a distinct context. However, no such studies have been conducted to date.

### Present Research and Hypothesis

Based on the limitations of the previous research, the current study will focus on two major issues: the relationship between Chinese parents’ theories of intelligence and paternal/maternal involvement, separately, as well as the effect of the congruence and discrepancy of Chinese parents’ theories of intelligence on paternal/maternal involvement, separately. Some researchers have argued that incremental and entity theory are two ends of one dimension of the intelligence theory spectrum (Blackwell et al., 2007; Claro et al., 2016; Haimovitz and Dweck, 2016), whereas others have suggested that the absence of an entity theory does not indicate the presence of an incremental theory (Jose and Bellamy, 2012; Shaari et al., 2017). We acknowledged the former in the current study. The originality of the study lies in analyzing potential differences between the maternal and paternal theories of intelligence and their contribution to parental involvement.

The previous research in this area usually assesses discrepancies with difference scores (univariate or multivariate), which have long been criticized for their questionable psychometric properties, such as unknown reliability and validity (Laird and Weems, 2011). Furthermore, it is hard to disentangle the effect of a difference score and the effects of the initial report variables on the outcome variable using the difference scores method (Edwards and Parry, 1993). In addition, traditional regression and difference scoring methods can only supply limited information (Wen et al., 2005; Edwards and Cable, 2009; Human et al., 2016). To better resolve the issues mentioned above, researchers utilize a method called response surface analysis (RSA). RSA has been used in generalization studies to describe nuanced relationships between two variables in a three-dimensional perspective (specifically, congruence and discrepancy with regard to outcomes). RSA is applied mainly to the study of outcomes of self-observed rating discrepancies in multisource feedback (Edwards and Parry, 1993) and is a straightforward approach that enables the simultaneous examination of the independent predictive ability of two perspectives as well as whether their congruence and discrepancy are consequential. Moreover, this approach could avoid the potentially problematic psychometric properties of the difference score method (Ostroff et al., 2005).

Therefore, in the current study, we use RSA to determine the relationship between parents’ theories of intelligence and parental involvement. RSA can examine how (a) the congruence, (b) the degree of the discrepancy, and (c) the direction of the discrepancy between father’s and mother’s theory of intelligence relate to paternal/maternal involvement. To summarize, RSA can provide the simultaneous and nuanced assessment of the united effects of paternal and maternal theories of intelligence on parental involvement within a single model (Cohen et al., 2010; Shanock et al., 2010).

In the current study, we conducted RSA twice, for paternal and maternal involvement as dependent variables separately, and used 1,694 matched pairs of Chinese parents as the object of our research. Based on previous studies, this study proposes the following assumptions: (1) fathers’ and mothers’ theories of intelligence relate not only to their own involvement but also to their involvement as a couple, and higher incremental theories are linked to higher levels of parental involvement; (2) the congruence of parents’ theories of intelligence relates to maternal and paternal involvement separately: specifically, higher incremental theories are linked with higher involvement; and (3) the discrepancy of fathers’ and mothers’ theories of intelligence relates to maternal and paternal involvement separately: specifically, the parent who holds a more incremental theory is more involved (the direction of discrepancy matters), and greater discrepancy between parents’ theories of intelligence is related to lower engagement (the degree of discrepancy matters).

## Materials and Methods

### Ethics Statement

All procedures in this study were approved by the Institutional Review Board of the Collaborative Innovation Center of Assessment toward Basic Education Quality, Beijing Normal University. Written informed consent to participate in the study was obtained from the parents of all the child participants before evaluation.

### Participants and Procedure

The study started with a total of 2,895 pairs of Chinese pupils’ fathers and mothers. We cooperated with the Education Bureau to conduct this survey. We selected 35 public primary schools from all the schools in Baoding City, Hebei Province, and 5 public primary schools in Beijing. A total of 1,951 fourth-grade students were from 48 classrooms in Baoding’s rural area, and 944 fourth-grade students were from 26 classrooms in Beijing. Beijing is the capital of China, and Hebei Province borders Beijing and is located in the middle-eastern part of China. Data were collected in December 2016. The parents’ questionnaires were taken home by the students, and the mothers and fathers completed the questionnaires at home separately. On the next day, the students returned the questionnaires to the school, where we collected them. Delayed questionnaires and receipts were sent back in one week.

The final sample included 1,694 pairs of mothers (27–54 years old, *M* = 36.46, SD = 3.93) and fathers (28–61 years old, *M* = 37.78, SD = 4.26) of Chinese pupils (864 boys, 830 girls; 9–11 years old, *M* = 9.39, SD = 0.50). We deleted samples based on the following rules: (1) questionnaires that were not answered by parents (588 pairs of parents deleted). In general, 79.69% of 2,895 pairs of questionnaires were answered by parents; (2) parents of students with intellectual disabilities, because the child’s disability might be related to parental involvement (Ferguson, 2002) (4 pairs deleted); (3) parents who did not live with their children, because parental involvement may be significantly lower if the parents and children live separately (Peña, 2000) (509 pairs deleted); and (4) parents for whom the deficiency rate of responses was above 1/3 (100 pairs deleted). The children belonging to the deleted questionnaires and the undeleted questionnaires had no significant differences in academic scores in the fall semester of 2016.

The mothers predominantly had a junior high-school education or technical secondary school education (41.6%), followed by a bachelor’s degree and above (18.4%), a middle-school education or secondary vocational school education (16.1%), a 3-year college education (12.8%), and a primary-school education or below (11.2%). Fathers also predominantly had a junior high-school education or technical secondary school education (47.2%), followed by a bachelor’s degree and above (18.5%), a middle-school or secondary vocational school education (14.6%), a 3-year college education (12.5%), and a primary-school education or below (7.2%). The annual disposable income of the family is calculated as the average of the mother-reported and father-reported data. Most of the students’ families (42.2%) had an average annual disposable income of 30,001–100,000 Chinese yuan; 9.9% had an income of below 7,200; 21.7% had an income of 7,201–30,000; 20.2% had an income of 100,001–300,000; and 6.0% had an income above 300,001. According to the China Statistical Yearbook [CSY] (2017), the median per capita annual income in China was 20,883 Chinese yuan, and the per capita net income was 52,530.4 Chinese yuan in Beijing and 19,725.4 in Hebei Province in 2016 (China Statistical Yearbook [CSY], 2017), which is approximately consistent with our data.

### Measures

#### Theories of Intelligence

The intelligence theory scale used was the Hong Kong version (Chen and Wong, 2014), a short version of the original questionnaire designed by Dweck (Dweck, 2000). The scale included 8 items, 4 that measured the incremental view and 4 that measured the entity view. An example item measuring the incremental view was “Everyone can significantly change his/her ability.” An example item measuring the entity view was “People can learn new things but cannot change their basic ability.” The parents rated each item from 1 (totally disagree) to 5 (totally agree). We reverse-scored the 4 items that measured the entity view and then calculated an average score of the total 8 items as the theory of intelligence score. Therefore, a higher total score indicates a higher incremental view, and a lower total score indicates a higher entity view. Cronbach’s α was 0.638 for the fathers’ reports and 0.664 for the mothers’ reports.

#### Parental Involvement

The 30-item scale of the “Parental involvement in primary-school children’s education questionnaire” (parent version) was formulated by Wu et al. (2013) (e.g., “I communicate with teachers regarding my child’s homework”) and was used by Wei et al. (2016). Fathers and mothers were separately asked about the frequency with which they performed each item during the last half of the year rating, using a 4-point Likert scale ranging from 1 (never) to 4 (usually). The average of the 30 items is the parent’s involvement score. Cronbach’s α was 0.928 for the mothers’ reports and 0.944 for the fathers’ reports.

#### Demographic Information

We collected demographic information from the schools and parents to rule out confounding variables. The schools provided the living settlement (urban or rural), children’s gender and age. Other demographic information, such as family disposable income and parents’ education level, was collected in the parents’ questionnaire, because previous studies have found that parental involvement is related to the child’s gender (Carter and Wojtkiewicz, 2000), the child’s age (Monique and Lefevre, 2002), the family living area and SES (Hickman et al., 1995).

### Data Analyses

We first cleaned the data with SPSS 22.0. All missing values (after deleting parents whose deficiency rate was above 1/3) were interpolated with the expectation-maximization (EM) estimation of missing data method (Allison, 2002). Then, we examined whether parental involvement varied with educational level, family disposable income, children’s gender and age and examined whether mothers’ and fathers’ theories of intelligence were related to parents’ involvement using Pearson correlation analyses.

After the preliminary analyses, we conducted an initial regression to investigate the pure relationship between one parent’s theory of intelligence and the other parent’s involvement. We included the father’s theory of intelligence and the mother’s theory of intelligence as independent variables in the regression and controlled the demographic variables for maternal and paternal involvement as dependent variables separately. To test multicollinear problems of variables, we performed a collinearity diagnosis. All the VIF values were below 10 (1.00–2.25), showing that there is no multicollinearity in the initial regressions (Neter et al., 1990).

Then, to prepare for RSA, multigroup latent variable modeling with Mplus 7.11 was conducted to ensure that the intelligence theory scale was equal for both mothers and fathers (Vandenberg and Lance, 2000).

Next, focusing on the discrepancy of the fathers’ and mothers’ reports, this study used the RSA method to analyze the data. RSA was conducted in three steps. First, we examined whether there were differences between mother- and father-reported theories of intelligence using standardized scores (Shanock et al., 2010). Then, to test for relationships between parents’ theory of intelligence and parental involvement, a polynomial regression was conducted. The general form of the polynomial regression is Z = b_0_ + b_1_X + b_2_Y + b_3_X^2^ + b_4_XY + b_5_Y^2^ + e, where Z is a dependent variable (maternal/paternal involvement), X is the mean-centered independent variable 1 (mother’s theory of intelligence), and Y is the mean-centered independent variable 2 (father’s theory of intelligence). Thus, the outcome variable is separately regressed on each of two independent variables (X and Y) with unstandardized beta coefficients b_1_ and b_2_, respectively, the interaction between the two independent variables (XY) with b_4_, and the squared term for each of the two independent variables (X^2^ and Y^2^) with b_3_ and b_5_, respectively.

We then evaluated the results with regard to four surface test values (a_1_–a_4_) (response surface pattern; Edwards and Parry, 1993) and examined the significance, which provided us with information on congruence and discrepancy. Values a_1_–a_4_ are derived from polynomial coefficients. Accordingly, a_1_ = (b_1_ + b_2_), a_2_ = (b_3_ + b_4_ + b_5_), a_3_ = (b_1_ - b_2_), a_4_ = (b_3_ - b_4_ + b_5_). We concentrate on whether (a) the congruence (a_1_, a_2_), (b) the degree of the discrepancy (a_3_), and (c) the direction of the discrepancy (a_4_) between two independent variables relate to dependent variables (Cohen et al., 2010; see Shanock et al., 2010 for a detailed explanation of the method). In the current study, we examined each of the four RSA coefficients to assess (1) a_1_: whether the parental involvement level has a relationship with parents’ theories of intelligence when the levels of the father’s and mother’s theories of intelligence are congruent; a significantly positive a_1_ illustrated that a high-level theory of intelligence related to a high level of involvement; (2) a_2_: whether the relationship of a_1_ is linear or nonlinear; a significant a_2_ represents a nonlinear relationship, and an insignificant a_2_ indicates a linear relationship; (3) a_3_: whether the direction of discrepancy in the theory of intelligence, such as when the level of one parent’s theory of intelligence is higher than that of the other, is related to parental involvement; a significantly positive a_3_ for the father shows that when the level of the father’s theory of intelligence is higher than the mother’s, paternal involvement is higher; and (4) a_4_: whether the degree of discrepancy in theory of intelligence is related to parental involvement; a significantly positive a_4_ indicates that more discrepancy relates to more involvement.

The corresponding graphical depictions of RSA help illustrate the nature of the effects by presenting the relationship in three-dimensional space. Specifically, the two lines in the figure reflect different combinations of the father’s and mother’s theories of intelligence, i.e., congruence versus discrepancy. The line from the corner where both parents’ theories of intelligence are low to the corner where both parents’ theories of intelligence are high is the line of congruence, namely, “the line of perfect agreement.” Coefficients a_1_ and a_2_ represent the slope and curvature of the line Y = X, respectively. The line from the corner where the father’s theory of intelligence is low and the mother’s theory of intelligence is high to the corner where the father’s theory of intelligence is high and the mother’s theory of intelligence is low is considered “the line of discrepancy.” Coefficients a_3_ and a_4_ represent the slope and the curvature of line Y = -X, respectively. Using the RSA coefficients and corresponding figures, therefore, we examined how the congruence and discrepancy of parents’ theories of intelligence relate to maternal and paternal involvement.

## Results

### Preliminary Analyses

The results of the Pearson correlation (two-tailed) with the means and standard deviations of the measures are shown in Table 1.

**Table 1:** Intercorrelations, means, and standard deviations.

Variables	1	2	3	4	5	6	7	8	9
1. Child’s age	–								
2. Child’s gender	-0.01	–							
3. Parents’ educational level	-0.51ˆ*	-0.03	–						
4. Family disposable income	-0.52ˆ*	-0.03	0.60ˆ**	–					
5. Living settlement	-0.05ˆ*	0.00	-0.69ˆ**	-0.55ˆ**	–				
6. Mother’s theory of intelligence	-0.04	-0.04	0.21ˆ**	0.22ˆ**	-0.15ˆ**	–			
7. Father’s theory of intelligence	0.02	-0.04	0.18ˆ**	0.20ˆ**	-0.18ˆ**	0.30ˆ**	–		
8. Maternal involvement	-0.02	-0.03	0.23ˆ**	0.22ˆ**	-0.23ˆ**	0.16ˆ**	0.08ˆ**	–	
9. Paternal involvement	0.03	-0.03	0.14ˆ**	0.06ˆ*	-0.17ˆ**	0.11ˆ**	0.14ˆ**	0.33ˆ**	–
M	9.39	1.49	3.17	5.38	1.62	3.31	3.30	2.53	2.34
SD	0.50	0.50	1.39	2.00	0.48	0.51	0.53	0.45	0.48
Observed range	9-11	1-2	1-6	1-10	1-2	1.25-5	1.5-5	1-4	1-4


**Note*. Parents’ educational level was represented by the highest level of education obtained by either parent. For example, when a mother had a junior high-school education and a father had a bachelor’s degree, we selected bachelor’s degree as the couple’s educational level. Living settlement was the family’s living area (1 = urban; 2 = rural). ^∗^*p* < 0.05. ^∗∗^*p* < 0.01 (two-tailed).*

As shown in Table 1, the child’s gender was not significantly correlated with any of the study variables. However, educational level, family disposable income, and living settlement were correlated with both theories of intelligence and involvement variables. Both maternal and paternal involvement are correlated with the father’s and mother’s theories of intelligence.

### Multigroup Latent Variable Modeling

The multigroup comparison result indicated that the mother’s and father’s responses to the same questionnaire regarding the theory of intelligence fit the factor mean invariance model (complete invariance model, χ^2^ = 592.47, *df* = 52, CFI = 0.91, TLI = 0.91, RMSEA = 0.08; Δ*df* = 2, Δχ^2^ = 0.32, Δ*p* > 0.5; see Byrne et al., 1989; Vandenberg and Lance, 2000; Cheung and Rensvold, 2002).

### Discrepancies in Mothers’ and Fathers’ Theories of Intelligence

The results of the examination of differences between mother- and father-reported theories of intelligence are shown below.

As seen in Table 2, only 38.9% of the 1,694 pairs of parents reached congruence in parents’ theories of intelligence; in other words, there were numerous discrepancies between the parents’ theories.

**Table 2:** Levels of parents over, under, and in agreement with theories of intelligence.

Theory of intelligence	*n* (%)
Mother > Father	527 (31.1)
Mother = Father	659 (38.9)
Mother < Father	508 (30.0)


### Initial Regression

First, we conducted linear regression twice to determine the pure relationship between parental theory of intelligence in pairs and parental involvement for maternal and paternal involvement, separately, as dependent variables. Due to the insignificance of the correlation between the child’s age/gender and parental involvement, we only considered family disposable income, parents’ educational level, and living settlement in the regression as control variables. We adopted hierarchical regression to control demographic variables by using the “enter” opinion in SPSS 22.0.

Partly consistent with the Pearson correlations, after controlling for the family disposable income, parents’ educational level, and living settlement, maternal involvement was positively related to the mother’s theory of intelligence (β = 0.06; *p* < 0.05); paternal involvement was related to both the mother’s theory of intelligence (β = 0.10; *p* < 0.01) and the father’s theory of intelligence (β = 0.09; *p* < 0.01).

### Polynomial Regression

We conducted polynomial regression twice to separately analyze maternal and paternal involvement as dependent variables. Control variables were family disposable income, parents’ educational level, and living settlement. We adopted the hierarchical regression to control demographic variables by using the “enter” option in SPSS 22.0. From the polynomial regression, we obtained five polynomial coefficients (b_1_–b_5_).

**FIGURE 1:**
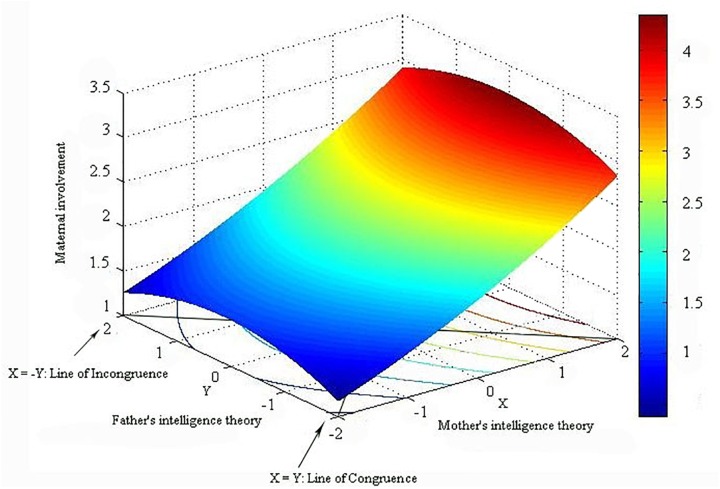
Three-dimensional results of the response surface of maternal involvement. *Note*. X-axis: level of mother’s theory of intelligence. Y-axis: level of father’s theory of intelligence. Z-axis: maternal involvement level.

**FIGURE 2:**
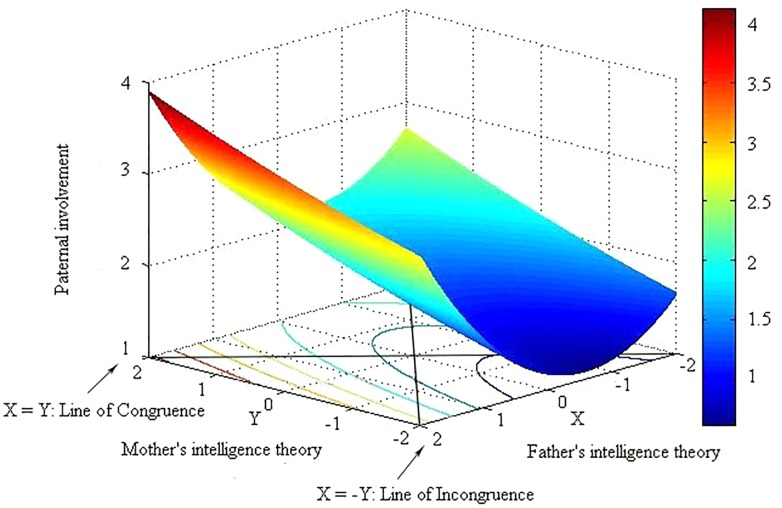
Three-dimensional results of response surface of paternal involvement. *Note*. X-axis: level of mother’s theory of intelligence. Y-axis: level of father’s theory of intelligence. Z-axis: paternal involvement level.

Then, we calculated four response surface coefficients (a_1_–a_4_) and significance to explore whether congruence and discrepancy between parents’ theories of intelligence were related to maternal and paternal involvement. These coefficients and their significance of polynomial regression and response surface are presented in Table 3. Further, the three-dimensional response surface of the relationship is provided in Figures 1, 2 as a visual illustration of the results.

**Table 3:** Initial regressions, polynomial regression and response surface results for parental theories of intelligence (IT) as independent variables of maternal and paternal involvement.

		Initial regression		Polynomial regression	
Dependent variables		Maternal involvement	Paternal involvement	Maternal involvement	Paternal involvement
β (SE)					
Constant		2.47 (0.09)^∗∗∗^	2.60 (0.09)^∗∗∗^	2.48 (0.09)^∗∗∗^	2.59 (0.09)^∗∗∗^
Family disposable income		0.02 (0.01)^∗∗^	-0.02 (0.01)^∗∗^	0.02 (0.01)^∗∗^	-0.02 (0.01)^∗∗^
Parents’ educational level		0.03 (0.01)^∗^	0.02 (0.01)	0.03 (0.01)^∗∗∗^	0.02 (0.01)^∗^
Living settlement		-0.10 (0.03)^∗∗∗^	-0.16 (0.03)^∗∗∗^	-0.10 (0.03)^∗∗∗^	-0.15 (0.03)^∗∗∗^
Mother’s IT (b_1_)		0.09 (0.02)^∗∗∗^	0.06 (0.02)^∗∗^	0.07 (0.02)^∗^	0.10 (0.03)^∗^
Father’s IT (b_2_)		-0.004 (0.02)	0.09 (0.02)^∗∗∗^	-0.01 (0.03)	0.03 (0.03)
Mother’s IT squared (b_3_)		-	-	0.01 (0.03)	0.01 (0.03)
Product of parents’ IT (b_4_)		-	-	0.06 (0.04)	-0.08 (0.04)^∗^
Father’s IT squared (b_5_)		-	-	-0.03 (0.03)	0.12 (0.03)^∗∗∗^
Model coefficients	R	0.28	0.22	0.29	0.24
	R^2^	0.08	0.05	0.08	0.06
	Adjusted R^2^	0.08	0.05	0.08	0.06
Surface test coefficients	a_1_	-	-	0.07^∗^	0.13^∗∗^
	a_2_			0.03	0.04
	a_3_			0.07^∗^	0.07
	a_4_			-0.07	0.20^∗∗∗^


**Note*. b_*1*_–b_*5*_ depict coefficients of the polynomial regression equation. a_*1*_–a_*4*_ depict coefficients of the response surface test. β = Unstandardized coefficients for variables. SE = Standard error. R^*2*^ is the cumulative variance accounted for. ^∗^*p* < 0.05. ^∗∗^*p* < 0.01. ^∗∗∗^*p* < 0.001 (two-tailed).*

### RSA Effects With Maternal Involvement

**FIGURE 3:**
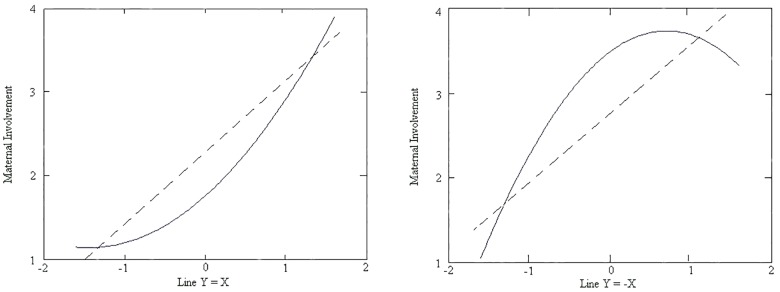
Transversal of the surface of maternal involvement above the line Y = X (left), transversal and the trend of the surface of maternal involvement above the line Y = –X (right).

The RSA plot (response surface) in which the dependent variable is maternal involvement is shown in Figure 1. In Figure 1, the X-axis represents the mother’s theory of intelligence, the Y-axis represents the father’s theory of intelligence, and the Z-axis represents maternal involvement. The lines Y = X and Y = -X vertically cut off the response surface, producing two curves that are shown in Figure 3. As shown in Table 3, regarding congruence, the a_1_ and a_2_ surface test coefficients indicated that maternal involvement has a significant relationship with the congruence of parents’ theories of intelligence. A significantly positive a_1_ indicates that when the father and mother hold the same level of theory, the stronger their beliefs are in the incremental theory they hold, and the higher maternal involvement will be (a_1_ = 0.07, *p* < 0.01). An insignificant a_2_ means that the relationship of congruent parental theories of intelligence and maternal involvement is linear (a_2_ = 0.04, *p* > 0.05). We can also see the trend from the near point to the remote point in Figure 1 and the dashed line in the left graph of Figure 3.

Next, we focused on whether the direction of discrepancy makes a difference (e.g., whether mother-reported theory of intelligence levels that are higher or lower than father-reported levels make a significant contribution to maternal involvement). A significantly positive a_3_ reflects that maternal involvement is higher when the discrepancy is such that the level of the mother’s theory of intelligence is higher than that of the father, as shown in the right corner of Figure 1, and higher than the left corner, where the level of the father’s theory of intelligence is high combined with a low level of the mother’s theory of intelligence (a_3_ = 0.07, *p* < 0.01). That is, when a mother holds a stronger incremental theory of intelligence than the father, maternal involvement is higher than when the mother holds a stronger entity theory of intelligence than the father. We can also see a trend from the left point to the right point in Figure 1. In the right graph of Figure 3, the trend of the transversal above line Y = -X is illustrated as a dashed line.

Finally, to investigate whether the degree of discrepancy between mother- and father-reported theory of intelligence matters, we calculated the a_4_ surface value. We found no relationship between the degree of discrepancy in parents’ views on intelligence and maternal involvement: a_4_ = -0.07, *p* > 0.05.

### RSA Effects With Paternal Involvement

**FIGURE 4:**
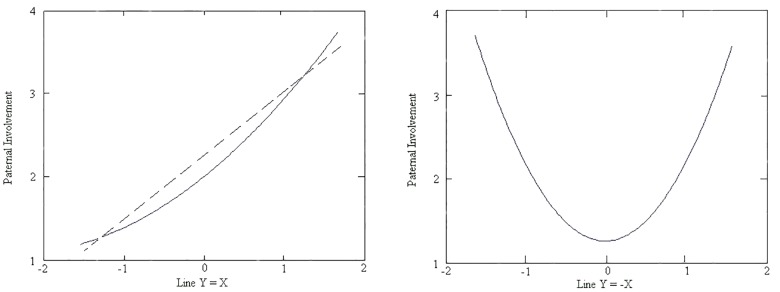
Transversal of the surface of paternal involvement above the line Y = X (left), transversal and the trend of the surface of paternal involvement above the line Y = –X (right).

The RSA plot (response surface) in which the dependent variable is paternal involvement is shown in Figure 2. In Figure 2, the X-axis represents the father’s theory of intelligence, the Y-axis represents the mother’s theory of intelligence, and the Z-axis represents paternal involvement. The lines Y = X and Y = -X vertically cut off the response surface, producing two curves that are shown in Figure 4. The results indicate that the congruence of parents’ theories of intelligence has a substantial effect on paternal involvement. A significantly positive a_1_ illustrates that when fathers and mothers hold the same level of theory, the incremental theory that they hold is stronger, and paternal involvement will be greater, similar to maternal involvement (a_1_ = 0.13, *p* < 0.01). Coefficient a_2_ is also insignificant, similar to the mother’s relationship; therefore, the relationship between congruent parental theories of intelligence and paternal involvement is linear (a_2_ = 0.03, *p* > 0.05). We can also see the trend from the right point to the left point in Figure 2 and the dashed line in the left graph of Figure 4.

Next, we focused on whether the direction of discrepancy created any difference in results. In contrast to maternal involvement, the direction of theory of intelligence has no relationship with paternal involvement. Coefficient a_3_ (the slope of line Y = -X) is not significant (a_3_ = 0.06, *p* > 0.05).

Finally, we examined whether the degree of discrepancy between mother- and father-reported theory of intelligence related to paternal involvement. A significantly positive a_4_ (a_4_ = 0.21, *p* < 0.01) indicates a U-shaped curve of paternal involvement on the line of Y = -X; in other words, paternal involvement would be significantly low when the mother’s theory of intelligence and the father’s theory of intelligence are congruent (the middle point of the line Y = -X, which is also the only point at which X = Y on the line Y = -X) and would increase significantly as the degree of discrepancy between the mother’s and father’s theory of intelligence increases (from the middle point to the two ends). As shown in Figure 4 (right), from left to right on the curve, paternal involvement first decreases, then increases, and then reaches the lowest point when it is in the middle of the line (where parental theories of intelligence are mostly in agreement), which means that paternal involvement is relatively high when father- and mother-reported theories of intelligence are incongruent. We can also see the trend from the near point to the remote point in Figure 2 and the right graph of Figure 4, which shows that paternal involvement is lowest when parental theories of intelligence reach agreement.

The results generally demonstrated that (1) when parental theories of intelligence were at the same level, both paternal and maternal involvement increased with the increase of parents’ incremental theory; (2) the direction of discrepancy of parental theories of intelligence was related to maternal involvement; and (3) and the degree of discrepancy of parental theories of intelligence was related to paternal involvement.

## Discussion

The current study aims to determine whether parental theories of intelligence relate to parental involvement as a couple, then focuses on the effect of the congruence and discrepancy between parental theories of intelligence on parental involvement separately.

The results first showed that the mother’s theory of intelligence was positively related to both paternal involvement and maternal involvement, while the father’s theory of intelligence was related only to paternal involvement. The congruence and discrepancy studies showed that, on the one hand, when parental theories of intelligence were at the same level, both paternal and maternal involvement increased with the increase in the level of parents’ incremental theory. On the other hand, when there was discrepancy between parental theories of intelligence, the direction of discrepancy was related to maternal involvement, and the degree of discrepancy was related to paternal involvement. Specifically, maternal involvement is higher when the discrepancy is such that the mother holds a stronger incremental theory than the father and is lower when the mother holds a stronger entity theory than the father; paternal involvement increased when the discrepancy between parental theories of intelligence was greater and decreased when the discrepancy was smaller.

### Parental Theories of Intelligence Together Are Related to Parental Involvement Separately

The current study found that maternal involvement was positively related to the mother’s theory of intelligence and that paternal involvement was positively related to the father’s theory of intelligence. Because a higher theory of intelligence score represents a more strongly held incremental theory, the results illustrated that a parent who holds a stronger incremental theory would desire to be more involved in their children’s education. This result is consistent with those of previous studies (Ames and Archer, 1987; Stipek et al., 1992; Aunola et al., 1999; Pomerantz and Dong, 2006; Moorman and Pomerantz, 2010). As discussed above, the underlying mechanism should be that parents who emphasize the role of effort motivate their children to accept their shortcomings and encourage them to think about the principle underlying tasks; thus, they are more involved in their children’s education. In contrast, when parents regard their children’s difficulties with learning as reflecting children’s low innate abilities, low levels of involvement occur.

In regard to the relationships between one parent’s involvement and the other’s theory of intelligence, the results are partly inconsistent with our hypothesis – paternal involvement was positively related to the mother’s theory of intelligence, whereas maternal involvement was not related to the father’s theory of intelligence. One explanation for this finding is the different roles that fathers and mothers play in family education. Based on the identity theory (Degarmo, 2010; Adamsons and Pasley, 2013), when an individual becomes a father or a mother, there are multiple social roles (such as breadwinner, daily caregiver, protector, etc.) that correspond to each parent. Individuals evaluate these roles according to social standards and social expectations and then form self-meaning identity criteria and behave consistently with these standards (Stryker and Serpe, 1994). In China, Confucianism provides a complete ethical system that draws distinctions for the gender equality standards: males are mainly responsible for the activities outside of the family (raising a family, earning the family income, etc.), while females deal with the affairs inside the family (raising the children, doing the housework, etc.) (Wu et al., 2013). When fathers and mothers both engage in the education of their children, this coparenting style reveals that mothers aim to control the educational activities via a practice known as maternal gatekeeping. Maternal gatekeeping is mothers’ preferences and attempts to restrict or encourage fathers’ involvement in their children’s care (Allen and Hawkins, 1999; Schoppe-Sullivan et al., 2008; Maddenderdich and Leonard, 2010; Zvara et al., 2013). The negative hindrance effect of mothers on fathers’ parenting is called the “gate-closing” effect, whereas the positive promotion effect of mothers on fathers’ parenting is called the “gate-opening effect” (Mcbride and Rane, 1998; Fox and Bruce, 2001; Maurer et al., 2001; Trinder, 2008). Similarly, another study has indicated that parents have had an influence each other’s parenting interactively (Xing et al., 2017), providing evidence for the existence of the gatekeeping effect in families.

In the current study, the gatekeeping theory might reasonably explain the observed results. Because mothers “control” the fathers’ engagement in education, paternal involvement is significantly positively related to the mother’s theory of intelligence, but maternal involvement is not related to the father’s theory of intelligence, which reflects the important position of the mother’s belief in family education. Moreover, such a “maternal gatekeeping” theory illustrates that maternal behaviors and attitudes might mediate paternal beliefs and involvement (Deluccie, 1995; Adamsons and Pasley, 2013). To further improve the understanding of “maternal gatekeeping” in the current study, we conducted mediation analysis. According to the theory of maternal gatekeeping, mothers may control fathers’ behavior based on the father’s beliefs (Gaunt, 2008). Therefore, we hypothesize the independent variable as the father’s theory of intelligence and the dependent variable as paternal involvement. The two potential mediators were the mother’s theory of intelligence and maternal involvement. Six regression analyses were then performed to test the potential mediators, and variables considered as covariates were controlled for in regression equations (Wen et al., 2005).

**FIGURE 5:**
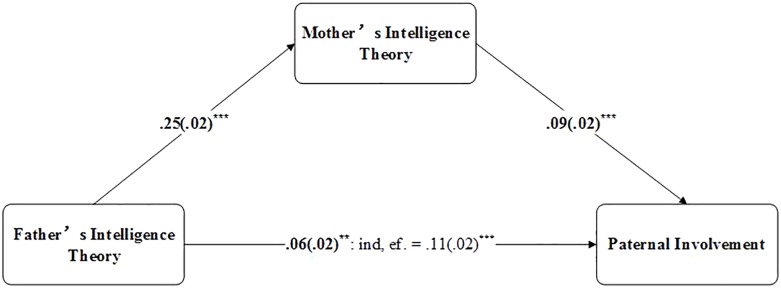
Summary of one distinct mediation model. *Note*. The independent variable was the father’s theory of intelligence, the dependent variable was paternal involvement, and the mediator was the mother’s theory of intelligence. The paths from the predictor to the dependent variable report the beta coefficient for the direct effects in bold characters and the indirect effects of the predictor (ind. ef.) with standard errors in brackets. ^∗^*p* < 0.05. ^∗∗^*p* < 0.01. ^∗∗∗^*p* < 0.001 (two-tailed).

The results showed that only the mother’s theory of intelligence is a partial mediator, as shown in Figure 5. The mother’s theory of intelligence partially mediated the relationship between the father’s theory of intelligence and paternal involvement, which supports the existence of maternal gatekeeping to some degree. Nonetheless, future study is needed to measure the impact of “maternal gatekeeping” using scales.

### Maternal-Paternal Congruence Is Related to Maternal and Paternal Involvement

In terms of the effect of congruence, as we speculated, we found that when the father and mother hold a theory at the same level, the incremental theory that they hold is stronger, the maternal and paternal involvement will be greater. These results are in accord with our hypotheses. As we speculated, because of the systematic nature of family, when family members’ beliefs are in agreement, the impact of the beliefs could increase parental interaction. Thus, under such circumstances, maternal and paternal involvement relate to the level of both maternal and paternal theories of intelligence.

On the one hand, the congruence of parents’ incremental theories of intelligence represents a positive educational environment in the family. As we theorized, when parents hold incremental theories, they concentrate more on the growth of their child’s ability, i.e., their child’s improvement with effort. Furthermore, when parents hold a theory at the same level (reach a state of congruence), the effect of their theories might improve due to the augmented interaction in the family. However, on the other hand, we must consider the negative aspect of congruence. Our results suggest that congruence may be harmful to involvement when both parents agree that ability is unchangeable. This congruence of high entity theory produces an inactive educational environment in the family; both the mother and father allow the child to pursue performance rather than learning, which could be even worse than the existence of discrepancies. If parents hold divergent beliefs, there is a chance that they may discuss this issue and find a way to resolve the problem, while complete agreement on an entity theory might lead to no discussion. A similar assumption was made in another study. Human et al. (2016) thought that high levels of congruence in the family could be problematic to children’s development, which serves as a warning.

### Maternal-Paternal Discrepancy Is Related to Maternal and Paternal Involvement

Regarding discrepancy, in the current study, we found only partial support for our hypotheses. The direction of discrepancy is important for maternal involvement. When the mother’s theory of intelligence is more incremental than the father’s theory, maternal involvement is higher than when the father’s theory of intelligence is more incremental than the mother’s. This discrepancy can also be explained by the different roles of mothers and fathers at home (Stryker and Serpe, 1994; Degarmo, 2010; Adamsons and Pasley, 2013; Wu et al., 2013), as discussed above. As the person responsible for education (Cowan and Cowan, 1988), the mother pays attention to the father’s theory of intelligence. When a mother who holds an incremental theory finds that the child’s father might hold an entity theory, to try to influence the father’s theory, she might engage more in education, thus demonstrating her theory to the child’s father. Consequently, the direction of the discrepancy was related only to maternal involvement.

With regard to the degree of discrepancy, paternal involvement increases as the degree of discrepancy increases, which might also be partly explained by the gatekeeping theory. When the level of the mother’s theory of intelligence is higher than the father’s (mother’s theory of intelligence is also higher than the zero point), the larger the discrepancy grows, and the more the mother will encourage father to engage in educational activities because the mother is “the expert of domestic work” in the family, thereby leading to increased paternal involvement (Allen and Hawkins, 1999). By contrast, when the mother’s theory of intelligence is lower than the father’s (the mother’s theory of intelligence is also lower than the zero point), maternal involvement will be less than the baseline value. In such circumstances, due to the lack of efficacy in involvement, mothers may therefore withdraw from educational activities, and consequently, they would no longer control the fathers’ parenting (Gaunt, 2008). Therefore, as the level of the father’s incremental theory increases, paternal involvement also increases, showing the positive relationship between the degree of discrepancy and paternal involvement.

### Limitations and Future Study

The limitations of our study provide directions for future studies. First, the current study is a cross-sectional study. Through the use of polynomial regression with RSA, we can determine only the correlation between parental theories of intelligence and parental involvement. Parental theories of intelligence relate to parental involvement (Hoover-Dempsey and Sandler, 1995, 1997; Moorman and Pomerantz, 2010; Muenks et al., 2015), and there might be an interaction between parental involvement and parents’ theories of intelligence. For example, a father who engages more in his children’s education may realize that his children’s ability could change through learning or training, which could change his theory of intelligence, thereby contributing to an increase in engagement. Therefore, a longitudinal or intervention study is necessary for the future.

Second, the dependent variable in the current study is the frequency of parental involvement, but the quality of parental involvement is also important. For example, some empirical evidence shows that parental theories of intelligence were related to the quality of parental involvement (Stipek et al., 1992; Moorman and Pomerantz, 2010). Thus, we should pay more attention to the quality of parental involvement. In future studies, we will research whether the congruence and discrepancy of fathers’ and mothers’ quality of parental involvement relate to parents’ theories of intelligence.

Third, the generalizability of this study to other populations is less clear. However, the objective of this study was to determine the relationship between parents’ theory of intelligence and parental involvement in Chinese culture. Future studies could investigate the relationship between parental theory of intelligence and parental involvement in other cultural contexts.

## Conclusion

In general, this study found the following: (1) the mother’s theory of intelligence related to both paternal and maternal involvement, while the father’s theory of intelligence related to paternal involvement only; (2) the congruence of parental theories of intelligence related to both paternal and maternal involvement; (3) the direction of discrepancy of parents’ theories of intelligence related to maternal involvement; and (4) the degree of discrepancy between parents’ theories of intelligence related to paternal involvement. To summarize, the current research described a detailed picture of the relationship between parents’ theories of intelligence and parental involvement; revealed the significance of the mother’s role in education; shed light on the importance of psychological factors in family members’ interaction; emphasized the importance of the congruence and discrepancy (direction and degree) between family members’ beliefs in educational processes; and contributed to the literature in developmental area by utilizing a rigorous statistical approach – RSA.

## Ethics Statement

This study was carried out in accordance with the recommendations of “the Institutional Review Board of the Collaborative Innovation Center of Assessment toward Basic Education Quality, Beijing Normal University” with written informed consent from all subjects. All subjects gave written informed consent in accordance with the Declaration of Helsinki. The protocol was approved by the Institutional Review Board of the Collaborative Innovation Center of Assessment toward Basic Education Quality, Beijing Normal University. Written informed consent to participate in the study was obtained from the parents of all the child participants before evaluation.

## Author Contributions

KJ: conception and design of the study and analysis and interpretation of the data. JL and CL: analysis of the data. XG and HZ: manuscript revision. BL, ZL, and LL: substantial contributions to the design of the study and final approval of the manuscript version to be published. Data collection was performed by all of the authors.

## Conflict of Interest Statement

The authors declare that the research was conducted in the absence of any commercial or financial relationships that could be construed as a potential conflict of interest.

## References

[B1] Adamsons K. Pasley K. (2013). Refining identity theory to better account for relationships and context: applications to fathering. *J. Fam. Theory Rev.* 5 159–175. 10.1111/jftr.12014

[B2] Allen S. M. Hawkins A. J. (1999). Maternal gatekeeping: mothers’ beliefs and behaviors that inhibit greater father involvement in family work. *J. Marriage Fam.* 61 199–212. 10.2307/353894

[B3] Allison P. D. (2002). Missing data: quantitative applications in the social sciences. *Br. J. Math. Stat. Psychol.* 55 193–196. 10.1348/000711002159653

[B4] Ames C. Archer J. (1987). Mothers’ beliefs about the role of ability and effort in school learning. *J. Educ. Psychol.* 79 409–414. 10.1037//0022-0663.79.4.409

[B5] Aunola K. Nurmi J. E. Onatsu-Arvilommi T. Pulkkinen L. (1999). The role of parents’ self-esteem, mastery-orientation and social background in their parenting styles. *Scand. J. Psychol.* 40 307–317. 10.1111/1467-9450.404131 10658515

[B6] Blackwell L. S. Trzesniewski K. H. Dweck C. S. (2007). Implicit theories of intelligence predict achievement across an adolescent transition: a longitudinal study and an intervention. *Child Dev.* 78 246–263. 10.1111/j.1467-8624.2007.00995.x 17328703

[B7] Bosma H. A. Jackson S. E. Zijsling D. H. Zani B. Cicognani E. (1996). Who has the final say? decisions on adolescent behavior within the family. *J. Adolesc.* 19 277–291. 10.1006/jado.1996.0025 9245283

[B8] Bowen M. (1966). The use of family theory in clinical practice. *Comp. Psychiatry* 7 345–374. 10.1016/S0010-440X(66)80065-2 5922263

[B9] Bowen M. (2010). Family therapy in clinical practice. *Fam. Process* 19 87–89. 10.1111/j.1545-5300.1980.087_1.x

[B10] Byrne B. M. Shavelson R. J. Muthén B. (1989). Testing for the equivalence of factor covariance and mean structures: the issue of partial measurement invariance. *Psychol. Bull.* 105 456–466. 10.1037/0033-2909.105.3.456

[B11] Carlson C. I. Cooper C. R. Sradling V. Y. (1991). Developmental implications of shared versus distinct perceptions of the family in early adolescence. *New Dir. Child Dev.* 51 13–32. 10.1002/cd.23219915103

[B12] Carter R. S. Wojtkiewicz R. A. (2000). Parental involvement with adolescents’ education: do daughters or sons get more help? *Adolescence* 35 29–44. 10841295

[B13] Chen W. Wong Y. (2014). What my parents make me believe in learning: the role of filial piety in Hong Kong students’ motivation and academic achievement. *Int. J. Psychol.* 49 249–256. 10.1002/ijop.12014 24990635

[B14] Cheung G. W. Rensvold R. B. (2002). Evaluating goodness-of-fit indexes for testing measurement invariance. *Struct. Equ. Model.* 9 233–255. 10.1097/NNR.0b013e3182544750 22551991 PMC3361901

[B19] China Statistical Yearbook [CSY] (2017). *China Statistical Yearbook.* Beijing: China Statistical Publishing House.

[B15] Cicognani E. Zani B. (1998). Parents’ educational styles and adolescent autonomy. *Eur. J. Psychol. Educ.* 13 485–502. 10.1007/BF03173100

[B16] Claro S. Paunesku D. Dweck C. S. (2016). Growth mindset tempers the effects of poverty on academic achievement. *Proc. Natl. Acad. Sci. U.S.A.* 113 8664–8668. 10.1073/pnas.1608207113 27432947 PMC4978255

[B17] Cohen A. Nahum-Shani I. Doveh E. (2010). Further insight and additional inference methods for polynomial regression applied to the analysis of congruence. *Multivariate Behav. Res.* 45 828–852. 10.1080/00273171.2010.519272 21103324 PMC2988441

[B18] Cowan C. P. Cowan P. A. (1988). Who does what when partners become parents: implications for men, women, and marriage. *Marriage Fam. Rev.* 12 105–131. 10.1300/J002v12n03_07 19342027

[B20] Degarmo D. S. (2010). A time varying evaluation of identity theory and father involvement for full custody, shared custody, and no custody divorced fathers. *Fathering* 8 181–202. 10.3149/fth.1802.181 20617120 PMC2898287

[B21] Deluccie M. F. (1995). Mothers as gatekeepers: a model of maternal mediators of father involvement. *J. Genet. Psychol.* 156 115–131. 10.1080/00221325.1995.9914811

[B22] Dweck C. S. (2000). Self-theories and goals: their role in motivation, personality, and development. *Nebr. Symp. Motiv.* 38 199–235. 10.1017/S0021963099226418 2130257

[B23] Dweck C. S. Leggett E. (1988). A social-cognitive approach to motivation and personality. *Psychol. Rev.* 95 256–273. 10.1037/0033-295X.95.2.256

[B24] Edwards J. R. Cable D. M. (2009). The value of value congruence. *J. Appl. Psychol.* 94 654–677. 10.1037/a0014891 19450005

[B25] Edwards J. R. Parry M. E. (1993). On the use of polynomial regression equations as an alternative to difference scores in organizational research. *Acad. Manag. J.* 36 1577–1613. 10.2307/256822

[B26] Egeren L. A. V. Hawkins D. P. (2004). Coming to terms with coparenting: implications of definition and measurement. *J. Adult Dev.* 11 165–178. 10.1023/b:jade.0000035625.74672.0b

[B27] Ferguson P. M. (2002). A place in the family. *J. Spec. Educ.* 36 124–131. 10.1177/00224669020360030201

[B28] Fox G. L. Bruce C. (2001). Conditional fatherhood: identity theory and parental investment theory as alternative sources of explanation of fathering. *J. Marriage Fam.* 63 394–403. 10.1111/j.1741-3737.2001.00394.x

[B29] Gaunt R. (2008). Maternal gatekeeping: antecedents and consequences. *J. Fam. Issues* 29 373–395. 10.1177/0192513X07307851

[B30] Haimovitz K. Dweck C. S. (2016). What predicts children’s fixed and growth intelligence mindsets? not their parents’ views of intelligence but their parents’ views of failure. *Psychol. Sci.* 27 859–869. 10.1177/0956797616639727 27113733

[B31] Hickman C. W. Greenwood G. Miller M. D. (1995). High school parent involvement: relationships with achievement, grade level, SES, and gender. *J. Res. Dev. Educ.* 28 125–134.

[B32] Hong Y. Y. Chiu C. Y. Dweck C. S. Lin D. M. S. Wan W. (1999). Implicit theories, attributions, and coping: a meaning system approach. *J. Pers. Soc. Psychol.* 77 588–599. 10.1037/0022-3514.77.3.588

[B33] Hoover-Dempsey K. V. Sandler H. M. (1995). Parental involvement in children’s education: why does it make a difference? *Teach. Coll. Rec.* 97 310–331.

[B34] Hoover-Dempsey K. V. Sandler H. M. (1997). Why do parents become involved in their children’s education? *Rev. Educ. Res.* 67 3–42. 10.2307/1170618

[B35] Hornby G. Lafaele R. (2011). Barriers to parental involvement in education: an explanatory model. *Educ. Rev.* 63 37–52. 10.1080/00131911.2010.488049

[B36] Human L. J. Melanie A. DeLongis D. A. Chen E. (2016). Congruence and discrepancy in adolescents’ and parents’ perceptions of the family: using response surface analysis to examine links with adolescents’ psychological adjustment. *J. Youth Adolesc.* 45 2022–2035. 10.1007/s109640160517-z 27287397

[B37] Huntsinger C. S. Jose P. E. (2009). Parental involvement in children’s schooling: different meanings in different cultures. *Early Child. Res. Q.* 24 398–410. 10.1016/j.ecresq.2009.07.006

[B38] Jose P. E. Bellamy M. A. (2012). Relationships of parents’ theories of intelligence with children’s persistence learned helplessness: a cross-cultural comparison. *J. Cross Cult. Psychol.* 43 999–1018. 10.1177/0022022111421633

[B39] Laird R. D. Weems C. F. (2011). The equivalence of regression models using difference scores and models using separate scores for each informant: implications for the study of informant discrepancies. *Psychol. Assess.* 23 388–397. 10.1037/a0021926 21319905

[B40] Li Z. (2007). Comparative analysis of family education in china and the united states and countermeasure research. *Contemp. Educ. Forum.* 138–139.

[B41] Lizza and Huang L. (2008). Comparison of family education between china and the united states. *Chin. Cult. Forum* 6 233–237.

[B42] Maddenderdich D. A. Leonard S. A. (2010). Parental role identity and fathers’ involvement in coparental interaction after divorce: fathers’ perspectives. *Fam. Relat.* 49 311–318. 10.1111/j.1741-3729.2000.00311.x

[B43] Maurer T. W. Pleck J. H. Rane T. R. (2001). Parental identity and reflected-appraisals: measurement and gender dynamics. *J. Marriage Fam.* 63 309–321. 10.1111/j.1741-3737.2001.00309.x

[B44] Mcbride B. A. Rane T. R. (1998). Parenting alliance as a predictor of father involvement: an exploratory study. *Fam. Relat.* 47 229–236. 10.2307/584971

[B45] McHale J. P. Kazali C. Rotman T. Talbot J. Carleton M. Lieberson R. (2004). The transition to coparenthood: parents’ prebirth expectations and early coparental adjustment at 3 months postpartum. *Dev. Psychopathol.* 16 711–733. 10.1017/S0954579404004742 15605633

[B46] Monique S. Lefevre J. A. (2002). Parental involvement in the development of children’s reading skill: a five-year longitudinal study. *Child Dev.* 73 445–460. 10.1111/1467-8624.00417 11949902

[B47] Moorman E. A. Pomerantz E. M. (2010). Ability mindsets influence the quality of mothers’ involvement in children’s learning: an experimental investigation. *Dev. Psychol.* 46 1354–1362. 10.1037/a0020376 20822244

[B48] Muenks K. Miele D. B. Ramani G. B. Stapleton L. M. Rowea M. L. (2015). Parental beliefs about the fixedness of ability. *J. Appl. Dev. Psychol.* 41 78–89. 10.1016/j.appdev.2015.08.002

[B49] Neter J. Wasseman W. Kutner M. H. (1990). *Applied Linear Statistical Models: Regression, analysis of Variance, and Experimental Designs*, 3rd Edn. Homewood, TL: Richard D. Irwin, Inc.

[B50] Ostroff C. Shin Y. Kinicki A. J. (2005). Multiple perspectives of congruence: relationships between value congruence and employee attitudes. *J. Organ. Behav.* 26 591–623. 10.1002/job.333

[B51] Peña D. C. (2000). Parent involvement: influencing factors and implications. *J. Educ. Res.* 94 42–54. 10.1080/00220670009598741

[B52] Pomerantz E. M. Dong W. (2006). Effects of mothers’ perceptions of children’s competence: the moderating role of mothers’ theories of competence. *Dev. Psychol.* 42 950–961. 10.1037/0012-1649.42.5.950 16953699

[B53] Schoppe-Sullivan S. J. Brown G. L. Cannon E. A. Mangelsdorf S. C. Sokolowski M. S. (2008). Maternal gatekeeping, coparenting quality, and fathering behavior in families with infants. *J. Fam. Psychol.* 22 389–398. 10.1037/0893-3200.22.3.389 18540767

[B54] Seginer R. (2006). Parents’ educational involvement: a developmental ecology perspective. *Parenting* 6 1–48. 10.1207/s15327922par0601_1

[B55] Shaari Z. H. Amar A. Harun A. B. Zainol M. R. (2017). Exploring the mindsets and well-being of rural secondary school students in Perak, Malaysia. *Glob. Bus. Manag. Res.* 9 728–737.

[B56] Shanock L. R. Baran B. E. Gentry W. A. Pattison S. C. Heggestad E. D. (2010). Polynomial regression with response surface analysis: a powerful approach for examining moderation and overcoming limitations of difference scores. *J. Bus. Psychol.* 25 543–554. 10.1007/s1086901091834

[B57] Shao X. Zhang J. (2010). Differences in family education views between china and the united states and intercultural communication studies. *Front. Econ. Cult.* 146–147.

[B58] Stipek D. Milburn S. Clements D. Daniels D. H. (1992). Parents’ beliefs about appropriate education for young children. *J. Appl. Dev. Psychol.* 13 293–310. 10.1016/0193-3973(92)90034-F

[B59] Stryker S. Serpe R. T. (1994). Identity salience and psychological centrality: equivalent, overlapping, or complementary concepts? *Soc. Psychol. Q.* 57 16–35. 10.2307/2786972

[B60] Tobin J. J. Wu D. Y. H. Davidson D. H. (1989). *Preschool in Three Cultures: Japan, China, and the United States.* New Haven, CT: Yale UniversityPress.

[B61] Trinder L. (2008). Maternal gate closing and gate opening in postdivorce families. *J. Fam. Issues* 29 1298–1324. 10.1177/0192513X08315362

[B62] Vandenberg R. J. Lance C. E. (2000). A review and synthesis of the measurement invariance literature: suggestions, practices, and recommendations for organizational research. *Organ. Res. Methods* 3 4–69.

[B63] Walker J. M. T. Wilkins A. S. Dallaire J. R. Sandler H. M. Hoover-Dempsey K. V. (2005). Parental involvement: model revision through scale development. *Elem. Sch. J.* 106 85–104. 10.1086/499193

[B64] Wang Q. Gao M. (2013). Comparison of family education between china and the united states. *Success* 6.

[B65] Wei W. Wu Y. Lv B. Zhou H. Han X. (2016). The relationship between parental involvement and elementary students’ academic achievement in china: one-only children vs. children with siblings 1. *J. Comp. Fam. Stud.* 47 483–500. 10.3138/jcfs.47.4.483

[B66] Wen Z. Hou J. Zhang L. (2005). Comparison and application of regulation effect and mediating effect. *Acta Psychol. Sin.* 2005 268–274. 9155652

[B67] Wu Y. Han X. Wei W. Luo L. (2013). The construction and verification of the theoretical model of parental education involvement in primary school students. *J. Beijing Norm. Univ.* 2013 61–69.

[B68] Xing S. Gao X. Song X. Archer M. Zhao D. Zhao M. (2017). Chinese preschool children’s socioemotional development: the effects of maternal and paternal psychological control. *Front. Psychol.* 8:1818. 10.3389/fpsyg.2017.01818 29093691 PMC5652336

[B69] Zhang S. Y. Carrasquillo A. L. (1996). Four Chinese junior high school ”unsuccessful” students: language, cultural, and family factors. *Acad. Fail.* 20.

[B70] Zhao Y. (2005). *Comparison of Chinese and American Family Education in Different Cultural Backgrounds.* Beijing: Beijing Education, 62–63.

[B71] Zvara B. J. Schoppe-Sullivan S. J. Dush C. K. (2013). Fathers’ involvement in child health care: associations with prenatal involvement, parents’ beliefs, and maternal gatekeeping. *Fam. Relat.* 62 649–661. 10.1111/fare.12023 26405366 PMC4578638

